# Effect of Working Temperature Conditions on the Autogenous Deformation of High-Performance Concrete Mixed with MgO Expansive Agent

**DOI:** 10.3390/ma16083006

**Published:** 2023-04-10

**Authors:** Zhe Cao, Zhongyang Mao, Jiale Gong, Xiaojun Huang, Min Deng

**Affiliations:** 1 College of Materials Science and Engineering, Nanjing Tech University, Nanjing 211816, China; 202061103063@njtech.edu.cn (Z.C.);; 2 State Key Laboratory of Material-Oriented Chemical Engineering, Nanjing Tech University, Nanjing 211800, China

**Keywords:** MgO expansive agent, autogenous shrinkage, temperature

## Abstract

Currently, mass concrete is increasingly utilized in various engineering projects that demand high physical properties of concrete. The water-cement ratio of mass concrete is comparatively smaller than that of the concrete used in dam engineering. However, the occurrence of severe cracking in mass concrete has been reported in numerous engineering applications. To address this issue, the incorporation of MgO expansive agent (MEA) in concrete has been widely recognized as an effective method to prevent mass concrete from cracking. In this research, three distinct temperature conditions were established based on the temperature elevation of mass concrete in practical engineering scenarios. To replicate the temperature increase under operational conditions, a device was fabricated that employed a stainless-steel barrel as the container for concrete, which was enveloped with insulation cotton for thermal insulation purposes. Three different MEA dosages were used during the pouring of concrete, and sine strain gauges were placed within the concrete to gauge the resulting strain. The hydration level of MEA was studied using thermogravimetric analysis (TG) to calculate the degree of hydration. The findings demonstrate that temperature has a significant impact on the performance of MEA; a higher temperature results in more complete hydration of MEA. The design of the three temperature conditions revealed that when the peak temperature exceeded 60 °C in two cases, the addition of 6% MEA was sufficient to fully compensate for the early shrinkage of concrete. Moreover, in instances where the peak temperature exceeded 60 °C, the impact of temperature on accelerating MEA hydration was more noticeable.

## 1. Introduction

The utilization of mass concrete has become increasingly prevalent in various civil engineering projects such as high-rise buildings, tunnels, and box girders, due to the upsurge in demand for such projects. Concrete is a fragile material with a lower tensile strength compared to its compressive strength, and mass concrete possesses the attributes of high total heat release and a large volume. The substantial size of the concrete impedes the timely dissipation of the heat generated from hydration, causing a rapid and considerable temperature increase in the mass concrete, with a significant temperature differential between the interior and exterior. Previously, dams utilized concrete with a high water-cement ratio and a relatively small amount of cement, leading to low heat of cement hydration and internal temperatures below 50 °C. However, in contemporary large-scale projects like retaining walls and bearing platforms, the utilization of mass concrete necessitates a smaller water-cement ratio, resulting in high heat of hydration and central temperatures above 70 °C, generating tensile stress that surpasses the concrete’s tensile limit, ultimately leading to cracking [1,2].

In the context of mass concrete with a low water-cement ratio, autogenous shrinkage is a crucial factor that contributes to cracking. In engineering, autogenous shrinkage is particularly pronounced in high-strength concrete, self-consolidating concrete, and mass concrete. Research indicates that high-strength concrete with a water-cement ratio of less than 0.3 can experience an autogenous shrinkage of 300–400 με and that the magnitude of autogenous shrinkage increases with the amount of cement per unit volume [3]. According to Erika Holt, the impact of the water-cement ratio on the autogenous shrinkage of concrete is generally insignificant when the ratio is greater than 0.4, but significant when it is less than 0.4 [4]. The autogenous shrinkage of concrete can be influenced by various factors such as the water-cement ratio, type of cement used, and mineral admixtures. According to Li’s investigation, the self-shrinkage of concrete without mineral admixtures is higher than that of concrete containing mineral admixtures. The self-shrinkage of mineral admixture concrete stabilizes after seven days, while non-admixture concrete continues to exhibit high levels of self-shrinkage for up to three weeks. While the self-shrinkage of pure cement is greater than that of mineral admixture concrete, it generates lower stress, and the cracking time of non-mineral admixture concrete is delayed [5]. Similarly, Lee’s research reveals that the self-shrinkage of slag concrete is greater than that of non-slag concrete, and the degree of self-shrinkage is directly proportional to the amount of slag present. This can be attributed to the fact that the particle size of slag concrete is finer than that of cement, resulting in a greater surface area, and denser pore structure following hydration. The chemical shrinkage value of concrete containing slag is higher compared to pure cement concrete [6]. A study conducted by Aveline revealed that while the autogenous shrinkage of slag cement concrete is higher, the time to cracking is longer than Portland cement concrete. However, under restrained conditions, slag cement concrete is more prone to cracking [7]. Laurent Barcelo’s investigation into the influence of SO_3_ content in cement on the dry shrinkage of concrete demonstrated that the dry shrinkage of concrete reduces as the SO_3_ content increases, as long as it does not surpass 3.1%. On the other hand, if it exceeds 3.1%, the trend is reversed [8]. Currently, there are several methods available for early concrete shrinkage measurement, including the volume method, length method, embedded sensor method, and other methods. Every approach has its own merits and demerits. When it comes to concrete, the volume method cannot be utilized due to the likelihood of aggregates causing harm to rubber bags. On the other hand, the length method is intricate and has a high probability of human errors. The most appropriate approach for measuring the initial deformation of concrete is through embedded sensors, although sine strain gauges are costly and non-reusable [9,10,11]. In this research, to precisely evaluate the early autogenous deformation of concrete, the embedded sensors are employed to determine the initial shrinkage of concrete.

Expansive agents are commonly utilized in dam engineering to counteract the notable shrinkage of mass concrete and to forestall cracking. The MgO expansive agent (MEA) is a particularly promising candidate due to its superior expansion performance and the adjustability of its expansion behavior [12,13]. In recent years, the application of MEA in practical engineering projects in China [14,15,16,17] has yielded systematic achievements in both the understanding of the expansion mechanism and the control of expansion amount [18,19] leading to the establishment of a comprehensive compensating system for mass concrete. The expansion performance of MEA is influenced by various factors, including the water-cement ratio, mineral admixtures, and curing temperature, with the latter being a significant determinant of MEA’s performance. Extensive research has confirmed that temperature exerts a profound impact on the reactivity of MEA. Specifically, a higher curing temperature accelerates the hydration rate of MEA, as evidenced by several studies [20,21,22]. Liu posits that the hydration process of mildly calcined MgO, akin to cement hydration, comprises five stages, including the initial, induction, acceleration reaction, deceleration reaction, and stable stages. The influence of temperature on the hydration reaction of MgO is significant, leading to pronounced temperature sensitivity in the expansion behavior of the MEA-cement slurry [23]. However, the hydration process of MEA in cement differs from that in pure form because of the competition for water between MEA and cement, leading to a significantly slower hydration rate of MEA in cement [24].

Given the high temperature sensitivity of both MEA and cement, investigating the impact of temperature on the deformation of MEA concrete is a crucial endeavor. There have been several investigations concerning the impact of curing temperature on the performance of MEA concrete. However, these studies were carried out mostly under conditions of high water-cement ratios and constant curing temperatures, which are inconsistent with the conditions encountered in large-scale vertical walls and load-bearing platforms where the temperature of the concrete can significantly increase due to the heat of hydration. Such engineering projects often require the use of a large number of cementitious materials and a relatively high water-cement ratio for dam concrete to achieve high concrete strength. Consequently, the central temperature of the concrete may reach over 70 °C, and the temperature is not constant. The use of constant temperature conditions in studying MEA concrete does not reflect the actual conditions encountered in engineering projects. To address this limitation, this study employs an insulation tool that can simulate the temperature change process of concrete under working conditions. By incorporating temperature and sine-type strain gauges into the concrete and monitoring the temperature and strain development of the concrete, the study aims to simulate the concrete strain in engineering structures and investigate the deformation performance of MEA concrete under different engineering temperatures.

## 2. Materials and Methods

### 2.1. Materials

**Figure 1:**
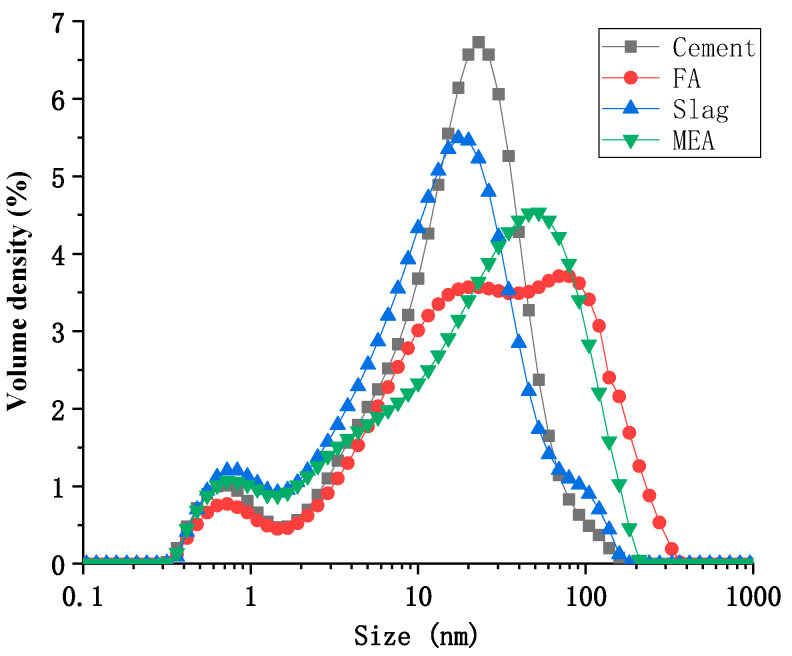
The particle size distribution curves of the materials.

In this study, ordinary Portland cement (OPC, P. II52.5) manufactured by Onoda Cement in Nanjing, China was employed. Fly ash (FA) was procured locally in Nanjing, China, while granulated blast furnace slag powder, designated as S95 mineral powder, was provided by Jiangsu Huailong Building Materials Co, Nanjing, China. The fine aggregate (fineness modulus of 2.7) had a well-graded size distribution and a well-rounded shape. Three types of coarse aggregate with the size grading of large size stone (20–25 mm), middle size stone (10–20 mm), and small size stone (5–10 mm) were used in the ratio of 2:5:3. The MEA used in the study was supplied by Jiangsu Subot, Nanjing, China, and was tested to have reaction values of 300 s using the citric acid method. The chemical composition, particle size, and specific surface area of the various adhesive materials used are presented in Table 1. It is noteworthy that the MEA had an average particle size of 22.76 μm. The particle size distribution curves of the materials are shown in Figure 1. The high-efficiency water-reducing agent (SP) used was polycarboxylate superplasticizer and was supplied by Jiangsu Subot, China. The water reduction rate of SP was determined to be 30%.

### 2.2. Tools for Simulating the Temperature of Working Conditions

**Figure 2:**
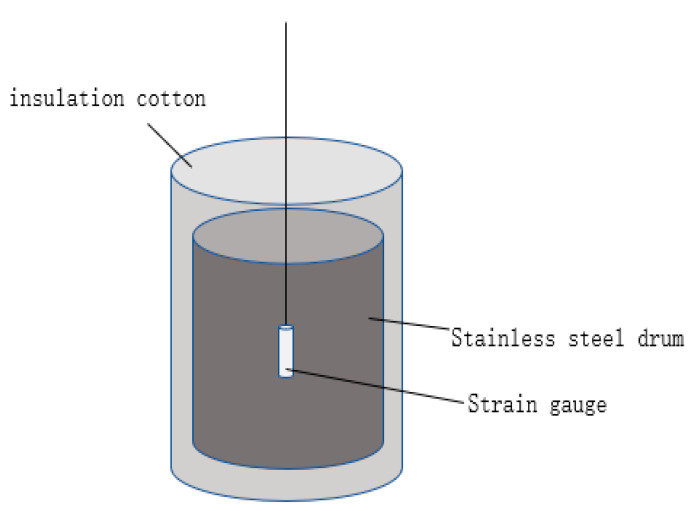
Schematic diagram of the simulation tool.

**Figure 3:**
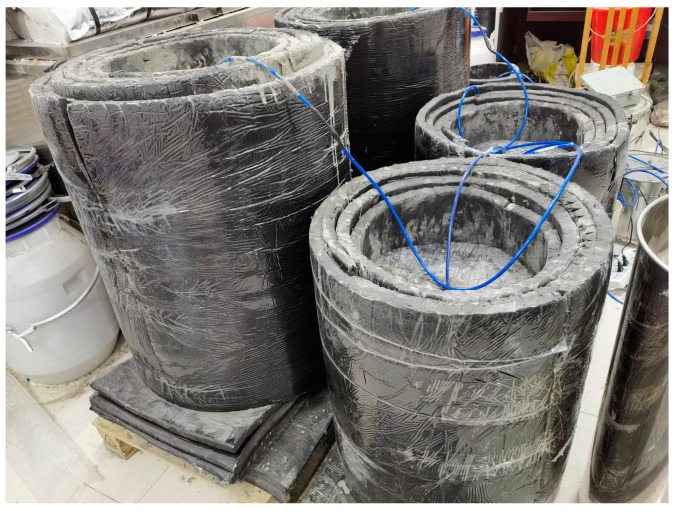
The simulation tools.

**Figure 4:**
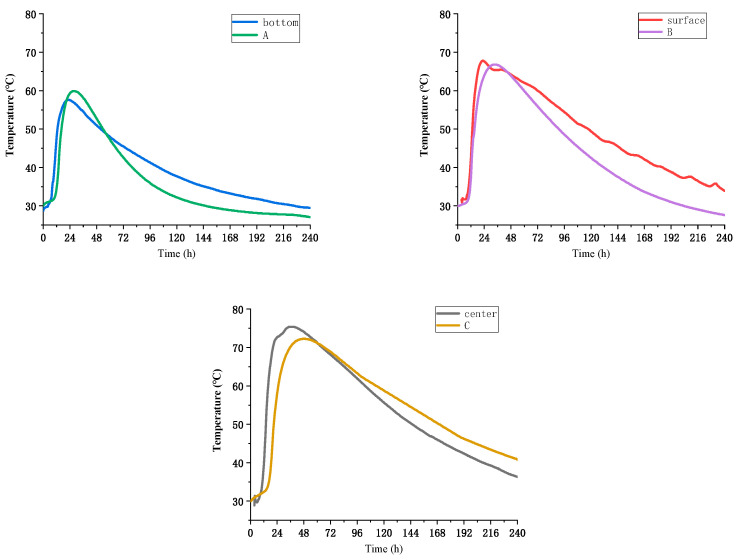
Comparison of the temperature rise of concrete in the simulation tools with the actual wall temperature.

This study aimed to develop tools that could simulate working conditions’ temperatures. The tools consisted of a stainless-steel barrel used as a container for the concrete, which was wrapped with insulation cotton around its exterior. The tool’s ability to simulate various temperature changes was achieved by adjusting the volume of the concrete and the thickness of the insulation layer and by selecting different insulation materials. Three tools were selected from the fabricated tools to match the actual temperature rise curve of the poured concrete. The simulation tools are visually represented in Figure 2 and Figure 3. The chosen insulation material is rubber and plastic thermal insulation cotton, with a thermal conductivity coefficient of 0.0302 W/m·K. The concrete volume and insulation layer thickness selected for the tools are shown in Table 2. A comparison of the temperature rise of concrete in the simulation tools with the actual wall temperature is presented in Figure 4. Temperature measurement was carried out by inserting a temperature sensor into the concrete. Temperature conditions were categorized based on the temperature peak, with the curve having the lowest temperature peak referred to as temperature condition A, the curve with a moderate temperature peak referred to as temperature condition B, and the curve with the highest temperature peak referred to as temperature condition C.

**Figure 5:**
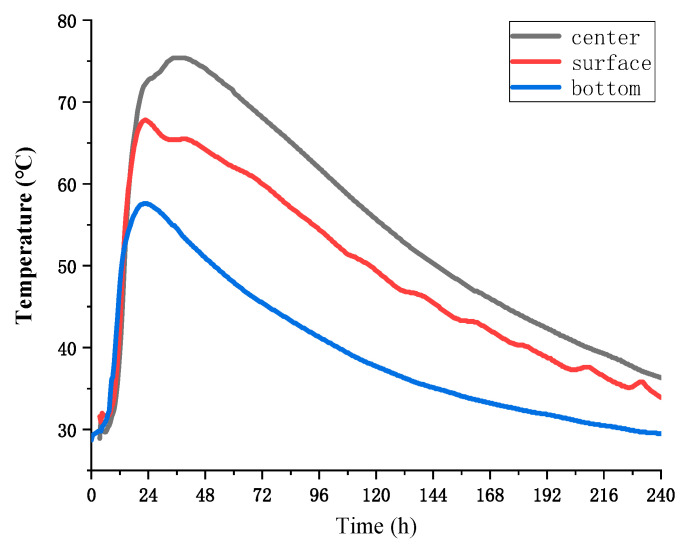
Temperature rise of the large thin wall.

To ensure that the simulation tools can effectively mimic the actual engineering structures, this study referred to the temperature-rise curve of a large thin-wall structure in a real project. The wall has a total length of 24 m, a height of 3.4 m, and a thickness of 1.1 m. The temperature rise was measured by using built-in strain gauges at the center, surface, and bottom of the wall, and the temperature rise of the wall is shown in Figure 5. The temperature rise at the center of the wall was the highest, reaching a peak temperature of 75.4 °C after 34 h of pouring; followed by the surface temperature rise, which reached a peak temperature of 67.8 °C after 22 h of pouring; the lowest temperature was at the bottom of the wall, reaching a peak temperature of 57.6 °C after 22 h of pouring.

### 2.3. Preparation of Concrete

Table 3 displays the mixture proportions of High-Performance Concrete (HPC) containing varying levels of MEA. The incorporation of MEA was achieved via internal admixture, at proportions of 0%, 6%, 8%, and 10% of the total cementitious material. The water-binder ratio for the concrete was set at 0.32. The manufacturing process involved weighing the raw materials in accordance with the mix proportions specified in Table 3, followed by the mixing of aggregates and binders in a mixer for one minute. Subsequently, water and a water-reducing agent were added to the mixer and stirred for five minutes. The dosage of polycarboxylate is 2.5% of the weight of the cementitious material. Following the mixing process, the concrete was poured into the simulation tools described in Section 2.2 for curing, in a completely dry state. The temperature rise is shown in Figure 5.

### 2.4. Test Methods

#### 2.4.1. Mechanical Properties

Based on the Chinese standard GB/T 50081-2019 [25], following the completion of the HPC mixing, the composite material was introduced into a mold with dimensions of 150 mm × 150 mm × 150 mm. Subsequently, the resulting specimens were transferred into a high-temperature maintenance box and subjected to incremental heating to replicate diverse temperature rise conditions for the compressive strength assessment. The compressive strength of the HPC samples for each mixture proportion at 3 d and 7 d was determined using a 2000 kN testing machine. To ensure the precision of the outcomes, three specimens were tested in each experimental group, and the mean compressive strength value of the three specimens was adopted as the final result.

#### 2.4.2. Autogenous Deformation

Temperature and Autogenous deformation of concrete at early ages were measured by using the tools described in Section 2.2, and three simulations were selected to simulate three temperature rise conditions. The tools were placed in a room with a constant temperature of 20 °C. The concrete was mixed with a mixer. After mixing, the concrete was vibrated with a vibrating table and then poured into the simulation tool with a built-in strain gauge to detect the temperature rise and strain of the concrete. The temperature and strain of the concrete changed rapidly in the early stage, so to be able to track them continuously, the strain gauges were tracked by an automatic measuring instrument manufactured by Ge Nan Industrial Co., Nanjing, China. The collection interval was 5 min. The total monitoring time was 14 days.

#### 2.4.3. Thermal Analysis

To evaluate the hydration extent of MgO, the thermogravimetric technique was employed on HPC mixtures lacking aggregate at the 3 d and 7 d stages in accordance with the Chinese standard GB/T 22314-2008 [26]. Following curing, the cement pastes were sectioned into small fragments, immersed in anhydrous ethanol for a minimum of 24 h to stop the hydration, and subsequently dried in a blast oven maintained at 60 °C for 24 h. The resultant small fragments were pulverized into a fine powder and filtered using a 0.075 μm square hole sieve. The powder was then subjected to heating from 30 °C to 1000 °C at a temperature ramp rate of 20 °C/min under an N2 atmosphere via the use of a TAQ600 synchronous thermal analyzer.

#### 2.4.4. SEM Morphology

To examine the microstructure of cement hydration products containing MEA, small portions of the cement pastes were sectioned, soaked in anhydrous ethanol for a minimum of 24 h to stop the hydration, and subsequently dried at 60 °C for 24 h. The resulting samples were then coated with gold and visualized using the Hitachi Regulus 8100 scanning electron microscope.

## 3. Results and Discussion

### 3.1. Mechanical Properties

**Figure 6:**
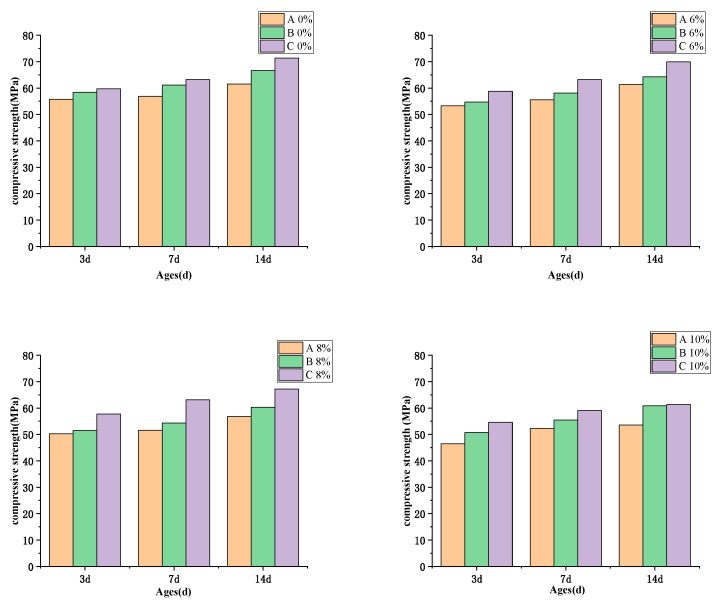
Effects of temperature conditions on the compressive strength of HPC mixed with MEA.

Figure 6 depicts the compressive strength of high-performance concrete (HPC) containing MEA at 3 day, 7 day, and 14 day intervals under three different curing temperatures, A, B, and C. The results indicate that an increase in MEA content causes a gradual decrease in the compressive strength of HPC. Specifically, when cured under temperature A, the compressive strength at 14 days decreased by 1.2 MPa, 4.7 MPa, and 7.9 MPa for HPC samples containing 6%, 8%, and 10% MEA content, respectively, in comparison to samples without MEA. Similarly, when cured under temperature B, the compressive strength at 14 days decreased by 2.3 MPa, 6.3 MPa, and 7.7 MPa for HPC samples containing 6%, 8%, and 10% MEA content, respectively, in comparison to samples without MEA. Additionally, when cured under temperature C, the compressive strength at 14 days decreased by 1.5 MPa, 4.2 MPa, and 10.1 MPa for HPC samples containing 6%, 8%, and 10% MEA content, respectively, in comparison to samples without MEA. These findings align with previous studies that indicate the addition of MEA has a detrimental effect on concrete compressive strength because MEA partially replaces cement in the binding material., leading to a reduction of calcium hydroxide and other essential materials that contribute to strength [27,28,29,30]. Furthermore, the weakened mechanical properties of the MEA HPC composite system could be attributed to the relatively diminutive crystal phase of the hydrated MgO products, shown in Equation (1), which possess inferior strength when compared to the cement hydration products. This fact implies that the strength of the system is significantly affected [31].

(1) $${Mg}^{2+}+2\;{OH}^{-}\to Mg{\left(OH\right)}_{2}$$

It is worth noting that the decline in strength observed at temperature C is relatively less pronounced compared to temperatures A and B, which could be attributed to the swift hydration of cement at high temperatures, which offsets the deleterious effects of MEA on the compressive strength of concrete.

### 3.2. Autogenous Deformation

**Figure 7:**
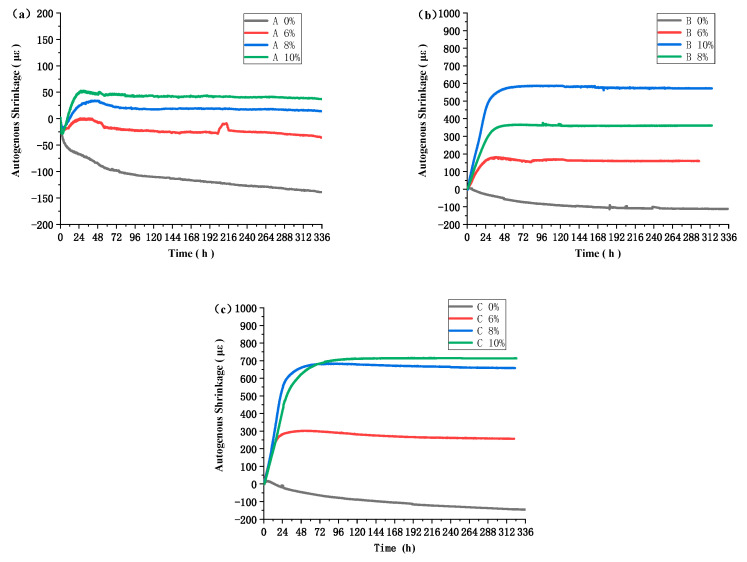
Effect of MEA content on early autogenous deformation of concrete at different temperature conditions. (**a**) Autogenous deformation at temperature A; (**b**) Autogenous deformation at temperature B; (**c**) Autogenous deformation at temperature C.

Figure 7 presents the autogenous shrinkage behavior of concrete with varying amounts of MEA content over a 14 day period, while subjected to similar temperature conditions. Concrete samples without MEA exhibited significant shrinkage at all three temperature conditions, with respective shrinkage values of 218.3 με, 111.6 με, and 145.7 με at temperatures A, B, and C. The samples demonstrated faster shrinkage rates prior to day 4, and the trend of shrinkage became relatively stable after day 4. As evident, higher MEA content resulted in greater expansion of concrete. Specifically, under temperature condition B, concrete samples containing 6%, 8%, and 10% MEA exhibited expansion rates of 159.7 με, 361.0 με, and 57.2 με, respectively. Similarly, under temperature condition C, concrete samples with MEA contents of 6%, 8%, and 10% exhibited expansion rates of 257.1 με, 657.9 με, and 713.3 με, respectively. It is important to note that at temperature condition A, adding 6% of MEA was not sufficient to fully offset the concrete shrinkage after 14 days. However, adding 8% of MEA at temperature condition A exhibited an adequate mitigating effect on shrinkage, while adding 6% of MEA proved effective at reducing shrinkage at temperature conditions B and C.

**Figure 8:**
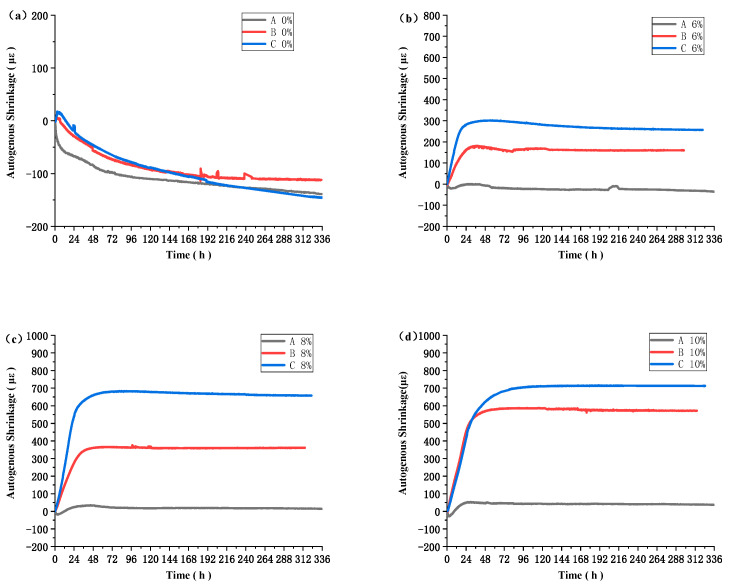
Effect of temperature on autogenous deformation of MEA concrete at early stage. (**a**) autogenous deformation, containing 0% MEA. (**b**) autogenous deformation, containing 6% MEA. (**c**) autogenous deformation, containing 8% MEA. (**d**) autogenous deformation, containing 10% MEA.

Figure 8 displays the impact of varying temperature conditions on autogenous deformation while maintaining a constant MEA content. The findings reveal that the performance of MEA improves with elevated concrete temperatures. In the case of concrete with 6% MEA content, it experiences a shrinkage of 37.37 με under temperature condition A, while it undergoes expansion under temperature conditions B and C, with expansion values of 159.7 με and 257.1 με, respectively. For concrete with 8% MEA content, under temperature conditions B and C, the expansion values increase by 55.7% and 183.8%, respectively, compared to condition A. Likewise, under temperature conditions B and C, the expansion values of concrete with 10% MEA content increase by 128.33% and 184.60%, respectively, compared to condition A. The effect of temperature on the performance of MEA has been established in previous studies, which have demonstrated that MEA undergoes a hydration process similar to cement, and its expansion value increases with higher curing temperatures [32].

### 3.3. Hydration Degree of MgO

**Figure 9:**
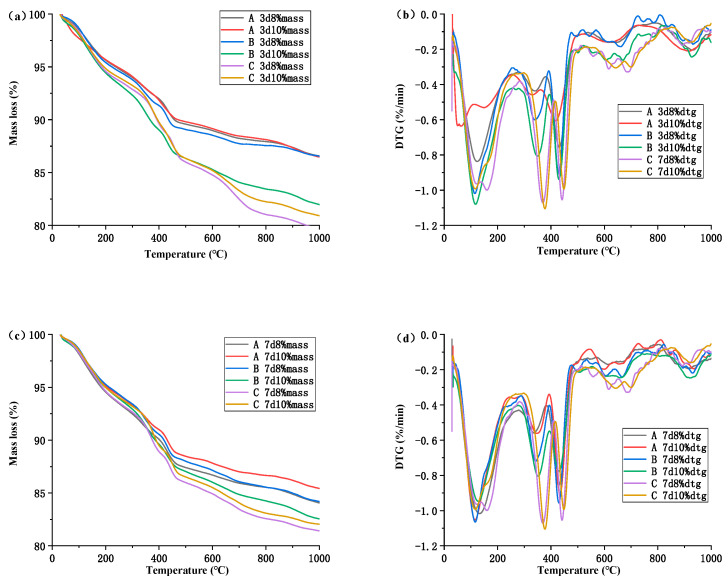
TG/DTG curves of the cement paste containing 8 wt% and 10 wt% MEA under different temperatures at 3 d and 7 d. (**a**) TG curves, at 3 d; (**b**) DTG curves, at 3 d; (**c**) TG curves, at 7 d; (**d**) DTG curves, at 7 d.

Figure 9 shows the TG/DTG curves of cement specimens mixed with MEA for 3 d and 7 d. As shown in Figure 9b,d, there are three different weight loss peaks on the DTG curve at 310–400 °C, 400–460 °C, and 650–750 °C, which correspond to the decomposition of Mg(OH)_2_, Ca(OH)_2_, and CaCO_3_, respectively. The degree of hydration of Mg(OH)_2_ and MgO(H MgO) was calculated using Equations (2) and (3), respectively, according to the literature [33,34].

(2) $$Mass\;{Mg(OH)}_{2}\;=\;\frac{58\times Mass\;loss(310\;{}^{\circ}C\sim 400\;{}^{\circ}C)}{18}$$

(3) $$H_{MgO}\;=\;\frac{40\times Mass\;loss\left(310\;{}^{\circ}C\sim 400\;{}^{\circ}C\right)}{n\times 18\times \left[1-Mass\;loss\left(950\;{}^{\circ}C\right)\right]}$$

where the Mass Mg(OH)_2_ represents the content of Mg(OH)_2_ formed in the hydrated cement pastes; the n represents the amounts of MEA in cement paste.

Table 4 illustrates the concentration of Mg(OH)_2_ and the extent of MgO hydration in cement paste that incorporates MEA. It can be observed that the addition of MEA results in an increase in the content of Mg(OH)_2_ in the samples, and a higher dosage of MEA corresponds to a greater degree of MgO hydration. For instance, under temperature condition A, the degree of MgO hydration after seven days was 32.41% and 38.19% when 8% and 10% of MEA were added to the cement, respectively. By comparing the degree of MgO hydration at three different temperature conditions, it can be inferred that the extent of MgO hydration escalates with an increase in the curing temperature. When the MEA concentration is 8%, the degree of MgO hydration at temperatures A, B, and C are 32.41%, 65.71%, and 84.34%, respectively. When the MEA concentration is 10%, the degree of MgO hydration at temperatures A, B, and C are 38.19%, 74.98%, and 84.40%, respectively. Evidently, higher curing temperatures facilitate the hydration of MgO. The degree of MEA hydration at temperature A is only 30%, whereas, at temperature B, it surpasses 60%. After seven days of hydration at temperature C, the degree of MEA hydration is around 80%. This implies that MEA hydrates expeditiously when the temperature exceeds 60 °C, and the rate of hydration does not increase proportionally with the temperature. The enhancing effect is relatively feeble before 60 °C.

### 3.4. Morphology

**Figure 10:**
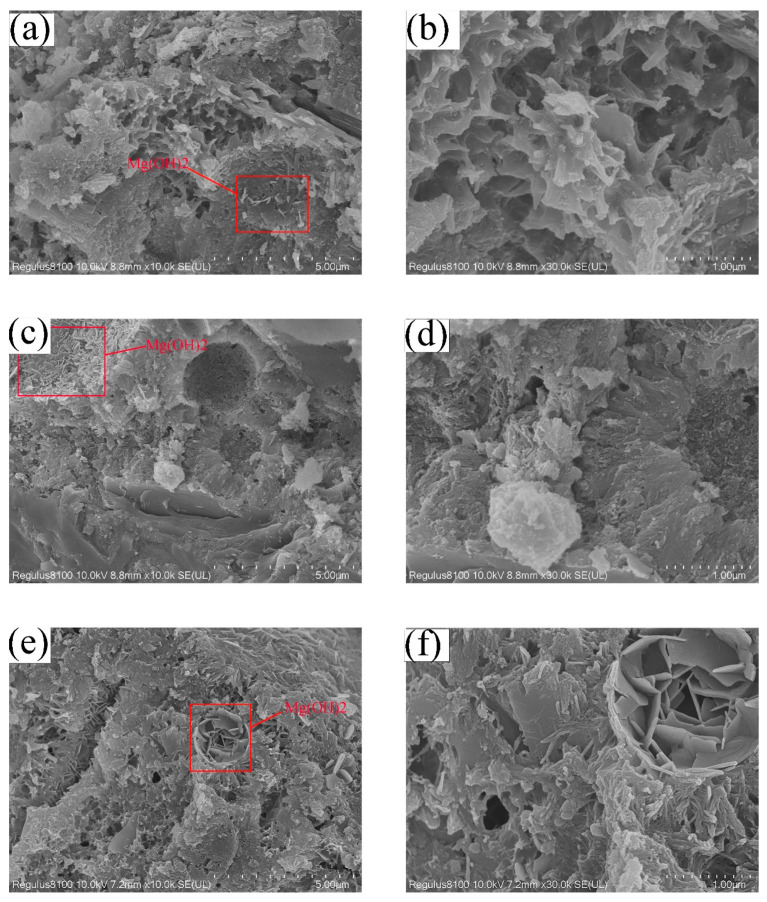
SEM images of hydration product in cement containing 8% MEA at 7 days (**a**) Microscopic morphology under temperature condition A; (**b**) Pore structure under temperature condition A; (**c**) Microscopic morphology under temperature condition B; (**d**) Microscopic morphology of Mg(OH)_2_ under temperature condition B; (**e**) Microscopic morphology under temperature condition C; (**f**) Microscopic morphology of Mg(OH)_2_ under temperature condition C.

Figure 10 exhibits the morphological changes that occur during the hydration of MEA in cement slurry at different temperatures. The formation of Mg(OH)_2_, which initiates at the surface of MEA particles and then progresses toward the center, is the primary product of this hydration process. An analysis of the figures indicates that the quantity of MEA hydration products increases with higher curing temperatures. For instance, in Figure 10a, the quantity of Mg(OH)_2_ is limited, and its observation is challenging. Conversely, Figure 10c depicts a substantial amount of Mg(OH)_2_ densely spread on the surface of spherical particles. Additionally, Figure 10e reveals the internal hydration of MEA particles, suggesting that MEA hydration is accelerated by higher temperatures. The figures also reveal that the density of the cement slurry increases with the curing temperature. Figure 10b clearly displays the pores generated during cement hydration, which are considerably reduced in Figure 10d,f.

### 3.5. Discussion

The impact of temperature on the performance of MEA is of great significance. The addition of MEA to concrete under temperature conditions B and C results in a larger expansion value compared to temperature condition A, which has a lower temperature peak. In temperature condition A, the early autogenous shrinkage of concrete cannot be fully compensated by the addition of 6% of MEA, while the addition of 8% of MEA only barely compensates for it. On the other hand, adding 6% of MEA at temperatures B and C leads to a significant expansion of the concrete. While the addition of 6%, 8%, and 10% of MEA does not differ much in expansion value at temperature A, there is a significant difference in expansion value at temperatures B and C with the addition of different amounts of MEA. Based on the thermogravimetry (TG) analysis, it is evident that the hydration of MEA is more complete at higher temperatures, which explains why the expansion of the concrete under temperature condition C is larger than under temperature conditions B and A. At temperature condition A, the hydration degree of MEA is only 30%, while at temperature condition B, the hydration degree of MEA has already reached over 60%. After 7 days of hydration at temperature condition C, the hydration degree of MEA is about 80%. This indicates that the hydration of MEA is rapid at temperatures above 60 °C, and the promoting effect is relatively weak before 60 °C, with the hydration rate not proportional to the temperature increase.

## 4. Conclusions

1. The effects of three different temperature conditions on the mechanical properties, autogenous shrinkage, and microstructure of high-strength concrete with MEA were studied. The main conclusions are as follows:
2. This investigation examined the impact of MEA incorporation on concrete’s compressive strength under different temperature conditions. The results demonstrate that the addition of MEA to concrete at different levels results in a decrease in compressive strength, and this reduction becomes more significant with increased MEA content. Notably, the decline in compressive strength at a higher temperature C is less pronounced than that observed in temperatures A and B. This outcome is attributed to the rapid hydration of cement at high temperatures, which offsets the detrimental effects of MEA on the compressive strength of concrete. This suggests that adding a higher amount of MEA could be considered in engineering processes where higher temperatures are involved.
3. In comparison to temperature condition A, which exhibits a lower temperature peak value, the use of MEA in concrete mixtures under temperature conditions B and C resulted in significantly greater expansion values. Under temperature condition A, the addition of 6% MEA failed to fully offset early autogenous shrinkage in the concrete. When 6% of MEA was used in mixtures under temperature conditions B and C, significant expansion was observed. While mixtures containing 6%, 8%, and 10% of MEA exhibited minimal differences in expansion values under temperature condition A, there were substantial differences in expansion values observed under temperature conditions B and C for mixtures containing varying amounts of MEA. In previous construction projects, the addition of 8% MEA has been a common approach to mitigate the impact of shrinkage on concrete stability. However, this study suggests that a lower concentration of 6% MEA is sufficient to compensate for the shrinkage in two out of three tested temperature conditions. Therefore, it may be worthwhile to reduce the amount of MEA in order to maintain the stability and strength of concrete.
4. MEA hydration is more complete at elevated temperatures, which accounts for the greater expansion of concrete at temperature C compared to temperatures A and B. At temperature A, MEA achieves only 30% hydration, while at temperature B, it achieves more than 60%. The degree of hydration for MEA after 7 days at temperature C is approximately 80%. MEA undergoes rapid hydration at temperatures exceeding 60 °C, and the rate of hydration is not directly proportional to temperature. Furthermore, the enhancing effect of temperature on the hydration of MEA is relatively weak before 60 °C.
5. This paper investigated the compressive strength and autogenous deformation of MEA concrete under three temperature conditions. However, the actual temperature rise of mass concrete in engineering applications is diverse. More temperature conditions should be studied. In addition, this study only used one type of MEA, and more types of MEA can be investigated in the future.

## Figures and Tables

**Table 1:** Chemical compositions and physical properties of the raw materials.

Chemical Composition	OPC	FA	SLAG	MEA
CaO (%)	65.32	3.8	44.06	1.88
SiO_2_ (%)	18.55	44.06	42.06	4.07
Al_2_O_3_ (%)	3.95	42.06	3.8	0.86
Fe_2_O_3_ (%)	3.41	2.91	0.57	0.57
MgO (%)	1.01	0.4	2.91	90.45
K_2_O (%)	0.72	0.49	0.49	/
Na_2_O (%)	0.18	/	0.75	/
SO_3_ (%)	2.78	0.75	/	/
LOI (%)	2.88	2.48	2.28	1.53
physical properties				
specific surface area (m^2^/g)	1.24	0.973	1.46	1.28
D50 (μm)	16.083	24.438	12.428	22.762

**Table 2:** The concrete volume and the thickness of insulation layer selected for the tools.

	Tool A	Tool B	Tool C
Concrete Vlome/m^3^	0.02	0.05	0.07
Thickness of insulation layer/cm	5	10	15

**Table 3:** Mix proportions of HPC matrix (kg/m^3^).

OPC	FA	Slag	MEA	Fine Aggregate	Coarse Aggregate			Water
					5–10 mm	10–20 mm	20–30 mm	
310	90	40	50	136	220	550	330	157
320	90	40	40	136	220	550	330	157
330	90	40	30	136	220	550	330	157
360	90	40	0	136	220	550	330	157

**Table 4:** Estimated quantity of Mg(OH)_2_ and hydration degree of MgO in cement pastes containing MEA.

	A 8%		A 10%		B 8%		B 10%		C 8%		C 10%	
	3 d	7 d	3 d	7 d	3 d	7 d	3 d	7 d	3 d	7 d	3 d	7 d
Mass loss at 320–400 °C/wt%	0.84	0.89	1.07	0.87	1.25	1.80	2.06	2.01	1.68	2.23	2.09	2.25
Mass Mg(OH)_2_/%	2.69	2.86	2.80	3.46	4.04	5.80	6.63	6.49	5.41	7.19	6.74	7.25
H MgO	30.44	32.41	31.29	38.19	44.54	65.71	67.17	74.98	74.76	84.34	79.48	84.40

## Data Availability

The data presented in this study are available on request from the corresponding author.
